# Supplementary material for: A structured additive modeling of diabetes and hypertension in Northeast India
Source: PLoS One. 2022 Jan 13;17(1):e0262560. doi: 10.1371/journal.pone.0262560 (PMC8758063; doi:10.1371/journal.pone.0262560)
Supplement: S1 Table — (PDF) [file pone.0262560.s001.pdf]

**S1 Table: Breakup of 82 districts by States in Northeast India.**

| State | Districts | State | Districts |
|---|---|---|---|
| **Arunachal Pradesh** | Tawang | **Assam** | Kokrajhar |
| | West Kameng | | Dhubri |
| | East Kameng | | Goalpara |
| | Papum Pare | | Barpeta |
| | Upper Subansiri | | Marigaon |
| | West Siang | | Nagaon |
| | East Siang | | Sonitpur |
| | Upper Siang | | Lakhimpur |
| | Changlang | | Dhemaji |
| | Tirap | | Tinsukia |
| | Lower Subansiri | | Dibrugarh |
| | Kurung Kumey | | Sivasagar |
| | Dibang Valley | | Jorhat |
| | Lower Dibang Valley | | Golaghat |
| | Lohit | | Karbi Anglong |
| | Anjaw | | Dima Hasao |
| **Nagaland** | Mon | | Cachar |
| | Mokokchung | | Karimganj |
| | Zunheboto | | Hailakandi |
| | Wokha | | Bongaigaon |
| | Dimapur | | Chirang |
| | Phek | | Kamrup |
| | Tuensang | | Kamrup Metropolitan |
| | Longleng | | Nalbari |
| | Kiphire | | Baksa |
| | Kohima | | Darrang |
| | Peren | | Udalguri |
| **Meghalaya** | West Garo Hills | **Manipur** | Senapati |
| | East Garo Hills | | Tamenglong |
| | South Garo Hills | | Churachandpur |
| | West Khasi Hills | | Bishnupur |
| | Ri Bhoi | | Thoubal |
| | East Khasi Hills | | Imphal West |
| | Jaintia Hills | | Imphal East |
| **Mizoram** | Mamit | | Ukhrul |
| | Kolasib | | Chandel |
| | Aizawl | **Tripura** | West Tripura |
| | Champhai | | South Tripura |
| | Serchhip | | Dhalai |
| | Lunglei | | North Tripura |
| | Lawangtlai | | |
| | Saiha | | |
