# Supplementary material for: A structured additive modeling of diabetes and hypertension in Northeast India
Source: PLoS One. 2022 Jan 13;17(1):e0262560. doi: 10.1371/journal.pone.0262560 (PMC8758063; doi:10.1371/journal.pone.0262560)
Supplement: Attachment — Submitted filename: Comments to authors_PONE-D-21-09955.docx [file pone.0262560.s002.docx]

The manuscript titled “A structured-additive modeling of diabetes and hypertension in Northeast India” addresses very important issue of diabetes and hypertension in India. The manuscript utilized the IDHS survey data and applied geo-additive logistic regression model to understand the influence of fixed effects and spatial heterogeneity. It also addresses the issue of non-linearity in spatial context. Overall, the work is important and study is conducted systematically to conclude about the findings.

There are some concerns which can be addressed before the manuscript can be accepted for publication

**Abstract**

L28: Rewrite the sentence as it is not clear.

L38: It can also be mentioned that why traditional linear regression models may fail to capture the spatial effects and why you choose Bayesian Geo-additive model. Very briefly, it can also be highlighted about the importance of accounting for spatial autocorrelation and how it can lead to bias in estimates.

L45, L46 and L47: The sentence can be re-written to make to clear about importance of unstructured effect for diabetes and structured effect for hypertension.

L49: It is not clear what you mean here by local and non-local factors.

L51: You mean to say here **should “be” given more importance**?

**Introduction**

L54 and L55: Reference can be added for these statements

L65-67: The statement can be re-written to make it clear

L67-69: Cite reference for this statement

L74: Sentence can be re-written a it appears you are mentioning about your findings in introduction.

L92: The reference cited (Ref No. 20) explores the availability of green spaces in neighborhood of individual households and it is not clear from your statement. It will be better to mention about the spatial scale here.

**Materials and Methods**

L112: Sentence is starting with abbreviation, kindly change it

L112: It can be mentioned in the reference when it was accessed/downloaded (Ref. No. 25)

L113: What necessary permissions were obtained? Please elaborate

L116: It can be made clear before this sentence that the survey done by IDHS and not done in this study to avoid confusion to readers

L122: There is some space in the total respondents which can be deleted – **112,062** and for **13,360**

L127: The breakup of 82 districts sampled for each state can be mentioned.

L129: Rewrite the sentence to make it clear

L134: OMRON is trade name so can be named appropriately

L146: The explanatory variables were selected from the IDHS survey? If so, mention how many variables were there in the IDHS database and how many were selected in this study? In addition, there is no mention about the continuous variables (BMI, wealth index and age) in your methods, but you present the results.

L155: It is not justified why you choose to use multiple regression model for narrowing down to the number of variables. As mentioned in comment for L146, you need to mention the total number of variables. You have left to readers to count the variables. There are many variable selection methods to reduce the number of variables before applying your Bayesian Geo-additive model. You need to justify your method or you have to perform variable selection method before narrowing down to the number of variables

L158: Again, mention about the number of variables which were used for fitting the Geo-additive logistic regression model

L164: You need to mention how you are defining your neighbor here? Did you use adjacency matric or any other method to define neighborhood, then you need to mention about it and may write the equation/formula for the same.

L169: It is advisable to use two more models here to understand the influence of spatially structure and spatially unstructured heterogeneity. The two models can be combination of M1 and M2 with spatial structured heterogeneity in one model and spatial unstructured heterogeneity in the other model. In this way it can be known about the drop/increase in DIC.

L189: How it was arrived to use 40,000 iterations? The convergence of different parameters should be tested using Gelman-Rubin statistics and mentioned that how these values were. You have not performed any cross validation statistics using CPO and PIT which is required to know the model performance.

L196: It should also be mentioned how you are deciding significance of variables based on 97.5% credible interval and values which do not bridge zero were not considered significant

**Results**

L199: The overall prevalence can also be shown on a map so that it can be compared with the spatial structured and unstructured heterogeneity maps for diabetes and hypertension

L237, Table 3: If this is the result of your M3 model which you say is combination of M1 and M2 then why the co-efficient of your continuous variables are not shown in this table? You have separately shown the non-linear effect of continuous variable in figures but this should also be reflected in your table or justify why it was not done so. It is not clear from your methods that whether your non-linear model using continuous variables also included categorical variables. It should be mentioned clearly to avoid confusion to readers. Your final model has all the variables which is my concern as mentioned in comment for L155 you need to reduce the total number of variables for your final model so that significant variables can be rightly identified.

L249, Table 4: same comments as for L237

**Discussion**

L300: You can start your discussion by saying about significant variables in your final model and then about the capturing non-linear effect using Geo-additive model

L327: You can refer your figures in discussion.

L350: This point contradicts your findings as mentioned on L246, that individuals who consume alcohol are at lower risk of hypertension. If this is the case only for Arunachal Pradesh or other states?

L358: Refer table number of your results

L359: It can be re-written with proper citation

L375 & 376: You mention previous studies, but cite only one reference.

L376: Need to add reference for this statement

L381-L383: It can be justified why you were constrained to use only IDHS data and why other variables were not included. You mentioned in several places about other variables like medical institutions, health care facilities, cost of living, urbanization and altitude may be playing a role in driving your spatial variability. It will be important to mention why you did not include other variables as this would have helped in even planning interventions and resource allocation in high risk areas for diabetes and hypertension

**Conclusion**

L395: As pointed out previously you need to mention clearly what you mean by local and non-local factors and preferable use other words to describe this pattern.
